# Endoplasmic reticulum oxidoreductin 1‐alpha deficiency and activation of protein translation synergistically impair breast tumour resilience

**DOI:** 10.1111/bph.15927

**Published:** 2022-08-11

**Authors:** Ersilia Varone, Alessandra Decio, Maria Chiara Barbera, Marco Bolis, Laura Di Rito, Federica Pisati, Raffaella Giavazzi, Ester Zito

**Affiliations:** ^1^ Istituto di Ricerche Farmacologiche Mario Negri IRCCS Milan Italy; ^2^ Institute of Oncology Research (IOR) Oncology Institute of Southern Switzerland Bellinzona Switzerland; ^3^ Bioinformatics Core Unit Swiss Institute of Bioinformatics Bellinzona Switzerland; ^4^ Histopathology Unit Cogentech S.C.a.R.L Milan Italy; ^5^ Department of Biomolecular Sciences University of Urbino Carlo Bo Urbino Italy

**Keywords:** breast cancer, endoplasmic reticulum stress, ERO1 alpha, ISRIB (integrated stress response inhibitor), PERK pathway, UPR (unfolded protein response)

## Abstract

**Background and Purpose:**

Endoplasmic reticulum (ER) stress triggers an adaptive response in tumours which fosters cell survival and resilience to stress. Activation of the ER stress response, through its PERK branch, promotes phosphorylation of the α‐subunit of the translation initiation factor eIF2, thereby repressing general protein translation and augmenting the translation of ATF4 with the downstream CHOP transcription factor and the protein disulfide oxidase, ERO1‐alpha

**Experimental Approach:**

Here, we show that ISRIB, a small molecule that inhibits the action of phosphorylated eIF2alpha, activating protein translation, synergistically interacts with the genetic deficiency of protein disulfide oxidase ERO1‐alpha, enfeebling breast tumour growth and spread.

**Key Results:**

ISRIB represses the CHOP signal, but does not inhibit ERO1. Mechanistically, ISRIB increases the ER protein load with a marked perturbing effect on ERO1‐deficient triple‐negative breast cancer cells, which display impaired proteostasis and have adapted to a low client protein load in hypoxia, and ERO1 deficiency impairs VEGF‐dependent angiogenesis. ERO1‐deficient triple‐negative breast cancer xenografts have an augmented ER stress response and its PERK branch. ISRIB acts synergistically with ERO1 deficiency, inhibiting the growth of triple‐negative breast cancer xenografts by impairing proliferation and angiogenesis.

**Conclusion and Implications:**

These results demonstrate that ISRIB together with ERO1 deficiency synergistically shatter the PERK‐dependent adaptive ER stress response, by restarting protein synthesis in the setting of impaired proteostasis, finally promoting tumour cytotoxicity. Our findings suggest two surprising features in breast tumours: ERO1 is not regulated via CHOP under hypoxic conditions, and ISRIB offers a therapeutic option to efficiently inhibit tumour progression in conditions of impaired proteostasis.

**What is already known**

- UPR in tumours fosters tumour cell survival and resilience to stress.

**What does this study add**

- The small molecule ISRIB interacts with deficiency of ERO1, enfeebling breast tumour growth and spread.

**What is the clinical significance**

- ISRIB can restrain the burden of breast tumours with limited ERO1 and high PERK.

**Abbreviations**
ATF4: activating transcription factor 4
BIP: binding immunoglobulin protein
CHOP: C/EBP homologous protein
eIF2alpha: eukaryotic initiation factor 2
ERO1 alpha: endoplasmic reticulum oxidoreductin 1 alpha
ISR: integrated stress response
ISRIB: inhibitor of integrated stress response
mTORC1: mammalian/mechanistic target of rapamycin complex 1
PDI: protein disulfide isomerase
PERK: PKR‐like endoplasmic reticulum kinase
RFS: relapse‐free survival
UPR: unfolded protein response

## 1 INTRODUCTION

Endoplasmic reticulum (ER) client proteins are folded and post‐translationally modified in the ER before being exported in the secretory pathway (Sun & Brodsky, 2019). The high rate of proliferation of cancer cells, together with cancer‐associated conditions such as hypoxia and shortage of nutrients, imposes stress on the ER, a process referred to as ER stress, which impairs its ability to fold and export proteins. As a consequence, a plethora of corrective measures are triggered, collectively known as UPR (Unfolded Protein Response), which increases resistance to stress and adaptation, and contributes to the thriving and survival of tumour cells (Cubillos‐Ruiz et al., 2017; Fels & Koumenis, 2006; Wang et al., 2012).

UPR, through its activated protein kinase RNA‐like endoplasmic reticulum kinase (PERK) branch, promotes the phosphorylation of eukaryotic initiation factor 2 alpha (p‐eIF2α) with consequent down‐regulation of global protein synthesis, thus reducing the protein load of the ER and relieving the stress. However, if protein synthesis restarts under conditions of impaired proteostasis, adaptation is shattered and cell death occurs (Han et al., 2013). Downstream from p‐eIF2α, ATF4, a pro‐survival factor, is selectively translated and leads to the transcription of genes involved in the ER functions (Guan et al., 2017). It also activates the transcription factor C/EBP homologous protein (CHOP). These two steps of attenuation of protein translation and ATF4 induction are also triggered by other pathways and therefore are part of the integrated stress response (ISR) (Guan et al., 2017).

CHOP regulates the expression of endoplasmic oxidoreductin 1 alpha (ERO1 α) (Marciniak et al., 2004). ERO1 α (henceforth ERO1) is a protein disulfide oxidase which, via PDI, introduces disulfide bonds in nascent proteins in the ER; thus, it is part of the adaptive UPR, favouring oxidative protein folding (Zito, 2015).

High levels of ERO1 are associated with different cancers and are predictive of their malignant phenotype and worse clinical outcome (Julian Cornelius et al., 2021; Tanaka et al., 2015; Tanaka et al., 2016; Yang et al., 2018; Zhang et al., 2020; Zhou et al., 2017). Recently, we and others characterized the potential for ERO1 to promote angiogenesis and breast cancer metastasis in hypoxia (Manuelli et al., 2021; May et al., 2005; Tanaka et al., 2016; Varone et al., 2021; Zilli et al., 2021). Our analysis of the secretome of breast tumour cells genetically deleted for ERO1 indicate that ERO1 promotes the secretion of different angiogenic factors, among them the master angiogenic regulator VEGF. Consequently, the inhibition of ERO1 in tumours could be a promising therapeutic strategy to impair angiogenesis and curtail tumour growth and metastasis (Varone et al., 2021).

Unfortunately, the currently available ERO1 inhibitors EN460 and QM295 suffer off‐target effects and prevent their use in vivo to test their ability to inhibit breast cancer growth and metastasis (Blais et al., 2010; Hayes et al., 2019). However, ISRIB, an inhibitor of p‐eIF2 alpha activity which rescues the repression of the protein translation, has no off‐target effects and its safety profile in preclinical cancer models suggests the possibility of using it in humans (Rabouw et al., 2019; Schoof et al., 2021; Sidrauski et al., 2015).

In this study, we set out to investigate whether ISRIB, by repressing p‐eIF2 alpha, which is upstream to ERO1 in the PERK branch of the ER stress response, also inhibits ERO1 activity, hence inhibiting tumour angiogenesis in breast cancer. Surprisingly, although ISRIB inhibited CHOP, it had no direct effect on either ERO1 expression or its angiogenic activity, suggesting that ERO1 expression is not regulated through CHOP in highly metastatic MDAMB231 breast tumours under hypoxic conditions. However, ISRIB, together with the genetic deficiency of ERO1, acts on the inhibition of protein translation, which is an adaptive feature of some tumours, and promotes proteotoxicity, thus synergistically limiting tumour growth.

## 2 METHODS

### 2.1 Cell lines

Cells were kept in culture for no more than 2 weeks and routinely tested for mycoplasma infection.

MDAMB231* cells were selected from parental MDAMB231(HTB‐26 from ATCC Frederick Cancer Tumour Repository, Maryland, USA) through passages in mice to enhance their tumorigenic and metastatic properties as described in Cruz‐Munoz et al. (2008). These cells were infected with a lentiviral vector carrying the coding sequence of the synthetic firefly luciferase gene, luc2 (*Photinus pyralis*). MCF7 cells (HTB‐22), parental MDAMB231 cells (HTB‐26), and HeLa cells (CRM‐CCl‐2) were purchased from ATCC. Primary cultures of endothelial cells (human umbilical vein endothelial cells (HUVECs) were isolated from umbilical cord veins (Jaffe et al., 1973) and grown in 1% gelatin‐coated flasks in M199 supplemented with 10% FBS, 10% newborn calf serum, 20 mM Hepes, 2 mM glutamine, 6 U·ml^−1^ heparin, 50 μg·ml^−1^ endothelial cell growth factor, penicillin, and streptomycin. Cells were used between the third and fifth passages.

### 2.2 Cell culture and transfection

Highly metastatic human MDAMB231* breast cancer cells were transfected with ERO1‐Lα CRISPR‐Cas9 KO plasmids (SC‐401747 for human, Santa Cruz Biotechnology) with three target‐specific guide RNAs (gRNA) of 20 nt. The plasmids were co‐transfected with homology‐directed repair HDR plasmids (SC‐401747‐HDR for human, Santa Cruz Biotechnology), which led to the insertion of a puromycin resistance gene and a red fluorescent protein (RFP) gene. Wild‐type, heterozygous and knock‐out clones are analysed by SDS‐PAGE and Sanger sequencing. ERO1 knock out (KO) HeLa cells and FLAG‐VEGF^121^ are described elsewhere (Varone et al., 2021).

### 2.3 Detergent‐insoluble and detergent‐soluble VEGF^121^

Detergent‐insoluble and ‐soluble VEGF^121^ and BIP were prepared as described earlier (Rai et al., 2021). FLAG Immunoblot was used to detect VEGF^121^. BIP was detected by a KDEL antibody.

### 2.4 Materials

Thapsigargin (Sigma‐Aldrich) and ISRIB (Selleckchem) were resuspended in DMSO at 5 mM. Paclitaxel (PTX, Indena S.p.A.) was dissolved in 50% Cremophor EL (Sigma‐Aldrich) and 50% ethanol and further diluted with saline before use. Salts and reagents were purchased by Sigma‐Aldrich.

### 2.5 Hypoxic chamber

Cells were transferred into a hypoxic chamber (Ruskinn Invivo2 400, UK) at 37°C and maintained in deoxygenated culture medium at the following gas concentrations: O_2_ 0.1%, CO_2_ 5% for 48 h. Control cells were maintained in standard culture medium in a normoxic incubator.

### 2.6 Motility assay

Conditioned media from equal numbers of WT and ERO1 KO MDAMB231* cells were used as an attractant to stimulate HUVEC migration. HUVECs were suspended in DMEM, 0.1% BSA at a concentration of 0, 75 × 10^6^ ml^−1^, and added to the upper compartment of Boyden chamber. The assay was carried out in 5% CO_2_ at 37°C for 6 h. At the end of the incubation, filters were fixed and stained with Diff‐Quik (Marz‐Dade, Dundingen, Switzerland) to detect cells adhering to the lower surface. Thereafter, migrated cells were counted in 10 high‐power fields for each filter.

### 2.7 Puromycin assay

1 × 10^6^ WT and ERO1 KO MDAMB231* were incubated 2 h with ISRIB at 200 nM and then resuspended in 100 μl of HMN buffer (50 mM HEPES pH 7.5, 150 mM NaCl and 2 mM MgCl_2_), with puromycin at a final concentration of 20 μg·ml^−1^ and incubated at 37C for 10 min. Afterwards, cells were resuspended in lysis buffer (Tris HCL pH 7.5 40 mM, EDTA 1 mM, EGTA pH 8 5 mM, and Triton 0.5%) with protease and phosphatase inhibitors; equal cell volumes (35 μl) were loaded onto a SDS PAGE gel. The puromycin signal was detected with a monoclonal puromycin antibody MABE343 (Merck Millipore). SUnSET western blotting was used to quantify the puromycin‐labelled peptides in WT and ERO1 KO cells treated with ISRIB. To demonstrate the specificity of the anti‐puromycin signal, a non‐puromycin treated sample was included. Ponceau staining of the membrane indicated equal loading of the total proteins among the samples. The western blots were quantified by acquiring the signal with ChemiDoc MP Imaging System and processing with Image Lab analysis software (Bio‐Rad Laboratories). In this quantification procedure, the puromycin signal of ISRIB‐treated WT and ERO1 KO cells was expressed relative to the mean puromycin signal in the sham samples.

### 2.8 MTS assay

Twelve thousand cells were incubated in MTS [3‐(4,5‐dimethylthiazol‐2‐il)‐5‐(3‐carboxymetoxyiphenil)‐2‐(4‐solfophenyl)‐2*H*‐tetrazolio] and PMS (Phenazine methosulfate), as indicated in the CellTiter 96® Aqueous Non‐Radioactive Cell Proliferation Assay (Promega). Acquisitions were made by TECAN infinite M200 with the excitation wavelength set at 490 nm.

### 2.9 Western blotting

Cells were lysed in cold buffer containing 150 mM NaCl, 20 mM HEPES pH 7.5, 10 mM EDTA and 1% Triton X100, and supplemented with a protease inhibitor cocktail (Roche), Phos Stop Easypack (Roche) and 20 mM NEM. Protein concentration was determined with a standard BCA assay (Pierce). Samples with the same protein concentration were mixed with non‐reducing Laemmli buffer (62.5 mM Tris–HCl pH 6.8, 2% SDS, 10% glycerol and 0.01% bromophenol blue) and heated for 5 min at 95°C. For reducing SDS‐PAGE, samples were supplemented with 100 mM DTT. Protein samples separated by either reducing or non‐reducing SDS‐PAGE were then transferred to Protran nitrocellulose membrane (Merck) and probed with the following antibodies: monoclonal mouse anti‐Actin (MAB1501, Sigma Aldrich), monoclonal mouse anti‐FLAG M2 antibody (F3165, Sigma‐Aldrich), monoclonal mouse anti‐KDEL (for BIP detection) (ADI‐SPA‐827, Enzo life Sciences) and polyclonal rabbit anti‐ERO1 alpha (Zito, Chin, et al., 2010), polyclonal p‐eIF2 alpha (44‐728G, Invitrogen) and eIF2 alpha (AH01182, Invitrogen). The western blots were quantified by acquiring the signal with ChemiDoc MP Imaging System and processing with Image Lab analysis software (Bio‐Rad Laboratories). The immuno‐related procedures used comply with the recommendations made by the *British Journal of Pharmacology*.

### 2.10 VEGF ELISA

Secreted VEGF was measured in the conditioned media of MDAMB231* cells with human VEGF Quantikine ELISA Kit (DVE00, R&D Systems).

### 2.11 Real‐time quantitative RT‐PCR analysis

Total RNA was isolated using the RNeasy Mini Kit (Qiagen) following the manufacturer's instructions. One microgram of total RNA was reverse‐transcribed and analysed using the Applied Biosystems' real‐time PCR System and the ΔΔCt method. Relative gene expression in cells was normalized to GAPDH or cyclophilin mRNA levels. The primer sequences are described in Varone et al. (2021).

### 2.12 Animals

Eight‐ to 10‐week‐old female SCID mice were obtained from Charles River Laboratories (Calco, Italy) and maintained under specific‐pathogen‐free conditions. SCID mice were housed in isolated vented cages, and handled using aseptic procedures. Animal studies are reported in compliance with the ARRIVE guidelines (Percie du Sert et al., 2020) and with the recommendations made by the *British Journal of Pharmacology* (Lilley, Stanford et al., 2020), in addition to the laws, regulations and policies governing the care and use of laboratory animals: Italian Governing Law (D. lgs 26/2014, authorization number 19/2008‐A issued 6 March 2008 by Ministry of Health; 395/2018PR to E. Zito); Mario Negri Institutional Regulations and Policies providing internal authorization for people conducting animal experiments (Quality Management System Certificate—UNI EN ISO9001: 2008—registration number 6121); the NIH Guide for the Care and Use of Laboratory Animals (2011 edition); EU directives and guidelines (EEC Council Directive 2010/63/UE), and in line with Guidelines for the welfare and use of animals in cancer research (Workman et al., 2010).

### 2.13 Breast tumour model and treatments

WT and ERO1 KO MDA‐MB231* cell suspensions were inoculated orthotopically (2 × 10^6^) in the mammary fat pad of 8‐ to 10‐week‐old SCID mice. The tumour volume was measured with a Vernier caliper once a week and calculated according to the formula *D × d2/2*, where *D* is the largest diameter of the tumour and *d* the smallest one. When tumours reached 50–100 mm^3^, two mice were killed for further analysis. G*Power, version 3.1.9.2, was used to calculate the power analysis to score differences in tumour growth. The other mice were randomized to receive vehicle (10 mice inoculated with WT cells and 10 mice with KO cells), ISRIB (10 mice inoculated with WT cells and 10 with KO cells), paclitaxel (10 mice inoculated with WT cells and 10 with KO cells) or the combination therapy with ISRIB and paclitaxel (10 mice inoculated with WT cells and 10 with KO cells).

Paclitaxel was injected intravenously (IV) at the dose of 15 mg·kg^−1^, Q7x2 (every 7 days for 2 weeks) and stopped 2 weeks before the end of the experiment. ISRIB (trans‐isomer, Aurogene s.r.l) was dissolved in 10% DMSO (Sigma‐Aldrich) in corn oil. It was injected interaperitoneally (IP), at the dose 2.5 mg·kg^−1^ every 2 days for 3 weeks.

Forty‐eight hours after the last dose of ISRIB (see Figure 3a, scheme of the pharmacological treatment), mice underwent volatile anaesthesia with isoflurane, analysed by bioluminescence imaging (BLI) and then killed by cervical dislocation. Metastases were quantified by BLI, where, mice injected with D‐luciferin (150 mg·kg^−1^ IP, Caliper Lifescience) were scanned after 10 min with IVIS Lumina Series III XRMS (Perkin Elmer). Images were analysed with the Living Image software (Perkin Elmer) and the metastasis burden was expressed as total flux (photons·s^−1^). The analyses were not blinded for practical constraints, because they were done by the same two researchers who performed the treatments. Primary tumours were randomly selected for further analysis: of which five for RNA sequencing and quantitative real‐time and five for histopathological analysis.

### 2.14 RNA sequencing

RNA was extracted from WT and ERO1 KO (vehicle, paclitaxel, ISRIB and the combination paclitaxel, ISRIB‐treated) xenografts (four separate samples for each condition) with the Qiagen RNeasy kit and quantified with Nanodrop; quality was measured using Qubit. The overall quality of sequencing reads was evaluated using FastQC (v.0.11.9). Sequence alignments of total‐RNA (stranded) to the reference human genome (GRCh38) were performed using STAR (v.2.7.9a) in two‐pass mode. Specifically, gene expression was quantified at the gene level by using the comprehensive annotations made available by Gencode (v38 GTF File). Samples were adjusted for library size and normalized with the variance stabilizing transformation in the R statistical environment using DESeq2 (v1.28.1) pipeline. When performing differential expression analysis between groups, we applied the embedded Independent Filtering procedure to exclude genes that were not expressed at appreciable levels in most of the samples considered. If not otherwise specified, all GSEAs were performed using the limma (v.3.44.3) package (Camera, use ranks set to FALSE). Gene‐set collections were retrieved from the Molecular Signature Database (MSigDB). *P*‐values were corrected for multiple testing using the false discovery rate (FDR) procedure, with the significance threshold set to 0.05. Data were deposited at European Nucleotide Archive (ENA) under accession number E‐MTAB‐11313.

### 2.15 Histopathology and immunohistochemistry

Subcutaneous breast tumours were collected and fixed in 10% neutral buffered formalin and paraffin‐embedded for histopathological analyses. To assess histological features Haematoxylin/Eosin (H&E) (Diapath) staining was performed according to standard protocol and samples were mounted in Eukitt (Bio‐Optica). The necrotic area was scored visually and the viable cells identified by the presence of nuclei. Anti CD31 (1:20, Abcam ab28364) was used to analyse tumoural angiogenesis.
For immunohistochemistry (IHC) analysis, paraffin was removed with xylene and the sections were rehydrated in graded alcohol. Antigen retrieval was carried out using a preheated target retrieval solution for 35 min. Tissue sections were blocked with FBS serum in PBS for 60 min and incubated overnight with primary antibody. The antibody binding was detected using a polymer detection kit (GAR‐HRP, Microtech) followed by a diaminobenzidine chromogen reaction (Peroxidase substrate kit, DAB, SK‐4100; Vector Lab). All sections were counterstained with Mayer's haematoxylin and visualized using a bright‐field microscope. Samples were analysed to count the CD31‐immunopositive areas.

### 2.16 Survival

Survival of breast cancer patients was analysed using the KMPlotter tool, which is publicly available at https://kmplot.com/analysis. (Dataset: Breast; Source: RNA‐Seq; Selected samples: All). Relapse‐free survival (RFS) was stratified into upper and lower quartiles according to either the gene expression levels of ERO1A, EIF2AK3 (PERK) or their ratio (EIF2AK3/ERO1A). Statistical significance was assessed using a log rank test.

### 2.17 Statistics

The data and statistical analysis comply with the recommendations of the *British Journal of Pharmacology* on experimental design and analysis in pharmacology (Curtis et al., 2018). Data are the mean ± SD, and were analysed with Prism 7 (GraphPad). Statistical analysis was based on the number of independent samples/experiments (n), as indicated in the figure legends. Statistical significance was evaluated using the unpaired *t*‐test for two‐group analysis, one‐way ANOVA or two‐way ANOVA for multiple comparison tests for three or more group analysis. Significant differences were set to *P* < 0.05. Results of Figures 2a–d and 4a,b were analysed by one‐way ANOVA followed by Sidak's multiple comparisons test. Tumour growth curve (Figure 3d,e) were analysed by two‐way ANOVA test, and as the *F* achieved statistical significance with Dunnett's multiple comparisons test. The constancy of variances was checked with Burtlett's test and, because the last time point showed no constant variance was corrected by the Geisser–Greenhouse test. Because lymph nodes and lung metastasis did not follow a normal distribution (D'Agostino & Pearson normality test), their values were expressed in log values and the non‐parametric Kruskal–Wallis test for multiple comparison was applied for the analysis. Further details of the statistics are reported in figure legends.

### 2.18 Nomenclature of targets and ligands

Key protein targets and ligands in this article are hyperlinked to corresponding entries in http://www.guidetopharmacology.org, and are permanently archived in the Concise Guide to PHARMACOLOGY 2021/22 (Alexander et al., 2021).

## 3 RESULTS

### 3.1 ERO1 deficiency impairs proteostasis and promotes the attenuation of protein translation in hypoxic conditions, which is counteracted by ISRIB

**FIGURE 1:**
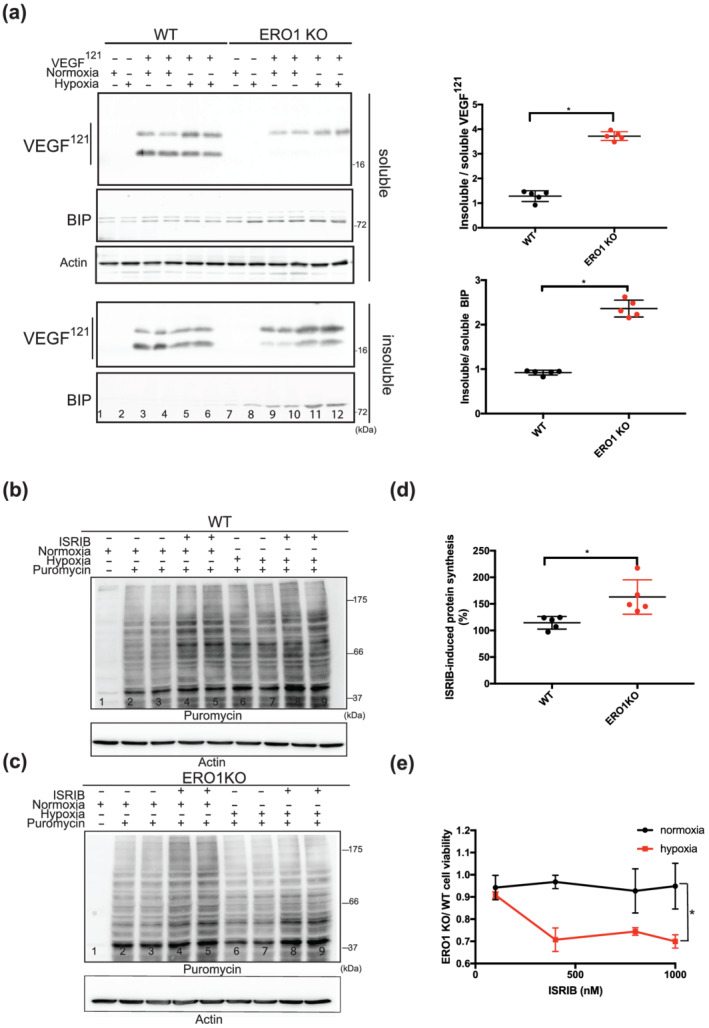
ERO1 deficiency promotes proteotoxicity and represses protein translation under hypoxic conditions, which is rescued by ISRIB. (a) Representative immunoblot of detergent‐soluble and ‐insoluble FLAG‐VEGF^121^ and BIP. Actin was used as a loading control. On the right, dot plots showing the ratio of detergent‐insoluble to detergent‐soluble VEGF^121^ and BIP. Ratio >1 indicates aggregates, and thus impaired proteostasis (n = 5, unpaired *t*‐test). (b) Representative immunoblot of newly synthesized, puromycin‐labelled proteins from WT MDAMB231* cells using an anti‐puromycin antibody. Actin was used as a loading control. (c) Puromycin labelling of proteins from ERO1 KO MDAMB231*. (d) Dot plots showing the percentage increase of the puromycin signal between ISRIB‐treated ERO1 KO and WT cells under hypoxic condition (n = 5, unpaired *t*‐test). (e) Ratio of the viability between ISRIB‐treated ERO1 KO and WT cells under normoxic and hypoxic conditions. Ratio less than 1 indicates impaired viability of ERO1 KO cells (n = 5 for WT and ERO1 KO cells at four different ISRIB concentrations, two‐way ANOVA).

Hypoxia is a common stress condition in solid tumours and their micro‐environment, and impairs post‐translational disulfide bond formation in nascent proteins in the ER (Koritzinsky et al., 2013). To gain insight into the impact of ERO1 loss on proteostasis under hypoxic conditions, we analysed the detergent‐soluble and ‐insoluble fractions of VEGF^121^, which contains intramolecular and intermolecular disulfide bonds (Iyer & Acharya, 2011). Remarkably, VEGF^121^, as well the chaperone BIP, was recovered more efficiently in the detergent‐insoluble fraction in ERO1 KO cells under hypoxic conditions and most likely in the aggregated unfolded state, indicating impaired proteostasis under these conditions (lanes 5–6 vs. 11–12 and dot plots of Figure 1a).

The increased BIP in ERO1 KO cells under hypoxic conditions suggested ER stress. ER stress triggers a homeostatic response, the so‐called UPR, which is involved in cancer thriving. One of the features of this ER stress‐related homeostatic response is to promote the attenuation of protein translation via p‐eIF2 alpha and through its PERK arm. In hypoxia, ERO1 KO cells had higher p‐eIF2 alpha indicating repression of the protein translation (Figure S1).

The two steps of attenuation of protein translation and selective ATF4 induction also are triggered by other pathways and are therefore part of the integrated stress response (ISR). Recently, ISRIB, an inhibitor of ISR, was shown to rescue p‐eIF2 alpha‐mediated attenuation of protein translation (Nguyen et al., 2018; Rabouw et al., 2019; Sidrauski et al., 2015). Furthermore, hypoxia induces a reduction in protein synthesis by activating the ISR arm of the UPR (Leprivier et al., 2015; Wouters & Koritzinsky, 2008). Therefore, to investigate the difference in this signalling between highly metastatic WT and ERO1 KO MDAMB231* breast cancer cells, we analysed their levels of protein translation in a puromycin‐based assay (SUnSET) (Schmidt et al., 2009) under hypoxia and with their response to ISRIB. WT cells were resistant to attenuation of protein translation in hypoxic conditions (lanes 6–7 vs. lanes 2–3, Figure 1b), whereas ERO1 KO cells experienced a reduction in protein translation (lanes 6–7 vs. lanes 2–3, Figure 1c), which was more efficiently recovered by ISRIB (lanes 6–7 vs. lanes 8–9, Figure 1c). These results suggest lower protein translation in ERO1 KO cells under hypoxia and a stronger recovery with ISRIB treatment (Figure 1d). Furthermore, a viability assay (MTS) pointed to reduced viability of ISRIB‐treated ERO1 KO cells, which had undergone hypoxia (Figure 1e).

These findings suggest that sustained activation of the adaptive arm of ISR, inducing attenuation of protein translation in proteostasis‐impaired ERO1‐devoid cancer cells under hypoxia, is counteracted by ISRIB, thus affecting their viability.

### 3.2 ISRIB does not inhibit ERO1 and the functionally‐related VEGFA signal

**FIGURE 2:**
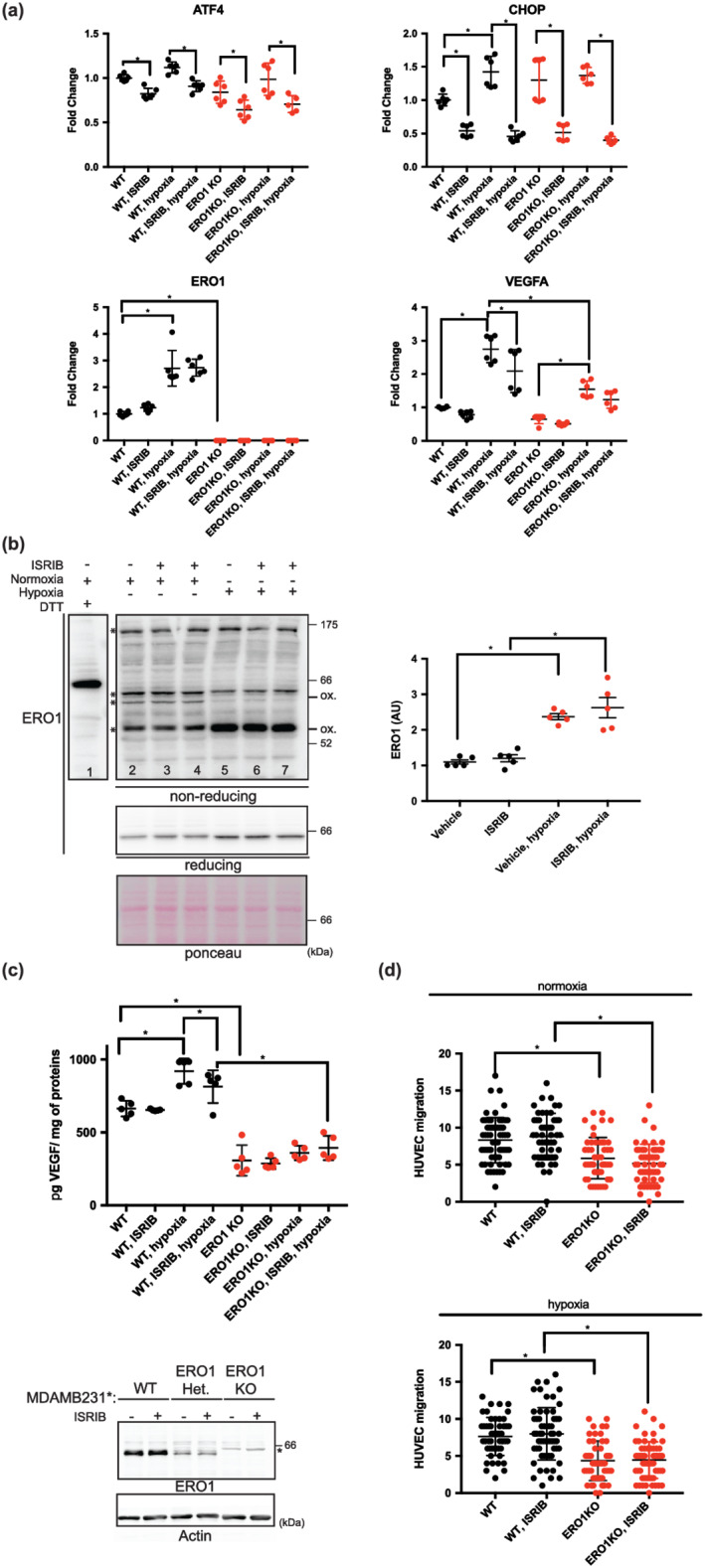
ISRIB inhibits ATF4 and CHOP signal but not that of ERO1. (a) Quantitative real‐time PCR on cDNA from WT and ERO1 KO MDAMB231* cells (n = 6). (b) ERO1 non‐reducing and reducing western‐blotting. Asterisks mark the different ERO1 bands in non‐reducing conditions; ox. indicates the oxidized ERO1. Ponceau indicates equal protein loading. On the right, dot plot indicating ERO1 levels in arbitrary units (AU) (n = 5). (c) ELISA of VEGF on conditioned media from WT and ERO1 KO MDAMB231* (n = 5). Below, ERO1 western blotting on WT, Het and ERO1 KO MDAMB231*, the asterisk indicates a background band. Actin indicates a loading control. (d) HUVEC migration assay using the conditioned media (CM) from equal numbers of WT and ERO1 KO MDAMB231* cultured in normoxic and hypoxic conditions as a chemoattractant. Differences were calculated by one‐way ANOVA for multiple comparisons.

Next, we examined whether ISRIB inhibits ERO1. ISRIB was reported both to reactivate protein translation and to inhibit ATF4, which is upstream of CHOP (Rabouw et al., 2019). CHOP also was repressed by ISRIB (Zyryanova et al., 2021). Since CHOP regulates ERO1, we wondered whether ISRIB inhibited ERO1 (Marciniak et al., 2004). Quantitative real time‐PCR confirmed the ISRIB‐mediated repression of ATF4, albeit very modestly, and CHOP transcripts in hypoxic treated WT MDAMB231* (Figure 2a) as well as in parental MDAMB231 and in the luminal MCF7 (Fig. Sup 2B).

However, we found no repressive effect on ERO1 at the level of mRNA (Figures 2a and S2) or levels of protein (Figure 2b). Non‐reducing ERO1 western blotting on protein lysate of WT MDAMB231* pointed to an increased level of one of ERO1 oxidized isoforms (ox.) in hypoxia, but, again, no difference was detected after ISRIB treatment, arguing against the possibility that ISRIB affects the ERO1 oxidative state, which reflects its activity (lanes 2–4 vs. lanes 5–7, Figure 2b) (Blais et al., 2010). Furthermore, quantitative analysis of the RNA levels indicated a reduction of VEGFA (the isoform mainly involved in tumour angiogenesis; Claesson‐Welsh & Welsh, 2013) in ERO1 KO cells under hypoxic conditions (Varone et al., 2021) (Figure 2a), but not that of VEGFB (Figure S2). Furthermore, we reported an up‐regulation of the VEGFA receptor VEGFR2 in ERO1 KO cells under hypoxic conditions (Figure S2), together with the repression of VEGFA in ISRIB‐treated WT cells (Figures 2a and S2).

Levels of secreted VEGFA in conditioned medium (CM) of MDAMB231* were impaired in ERO1 KO in normoxic conditions, inasmuch as the increase in VEGFA levels of WT was suppressed in ERO1 KO MDAMB231* under hypoxic conditions. In addition, ISRIB slightly impaired VEGF levels from WT but not those of ERO1 KO cells under hypoxia (Figure 2c).

To tackle the impact of ISRIB on the levels of secreted VEGF and its ability to promote angiogenesis, we exploited CM collected from equal numbers of WT and ERO1 KO MDAMB231* cultured in normoxic and hypoxic conditions, and treated or not with ISRIB to induce migration of HUVECs, which are primary endothelial cells with pro‐angiogenic potential. We confirmed our previous findings that showed a lower angiogenic potential of ERO1 KO MDAMB231* CM from normoxic conditions, but we could not detect any angiogenic effect of ISRIB in both normoxic and hypoxic conditions (Figure 2d). These results suggest that ISRIB impairs CHOP but has no effect on either ERO1 levels or ERO1‐related angiogenic activity.

### 3.3 ISRIB selectively impairs the growth and spread of ERO1‐deficient breast cancer

The detrimental effect of ISRIB on ERO1 KO MDAMB231* cells prompted us to investigate the therapeutic potential of ISRIB in the model of MDAMB231* breast cancer. We examined the growth of WT and ERO1 KO MDAMB231* breast tumours in immune‐deficient SCID mice treated with ISRIB, or with paclitaxel (PTX, which is the first line therapy for breast cancer), or the combination of the two.

**FIGURE 3:**
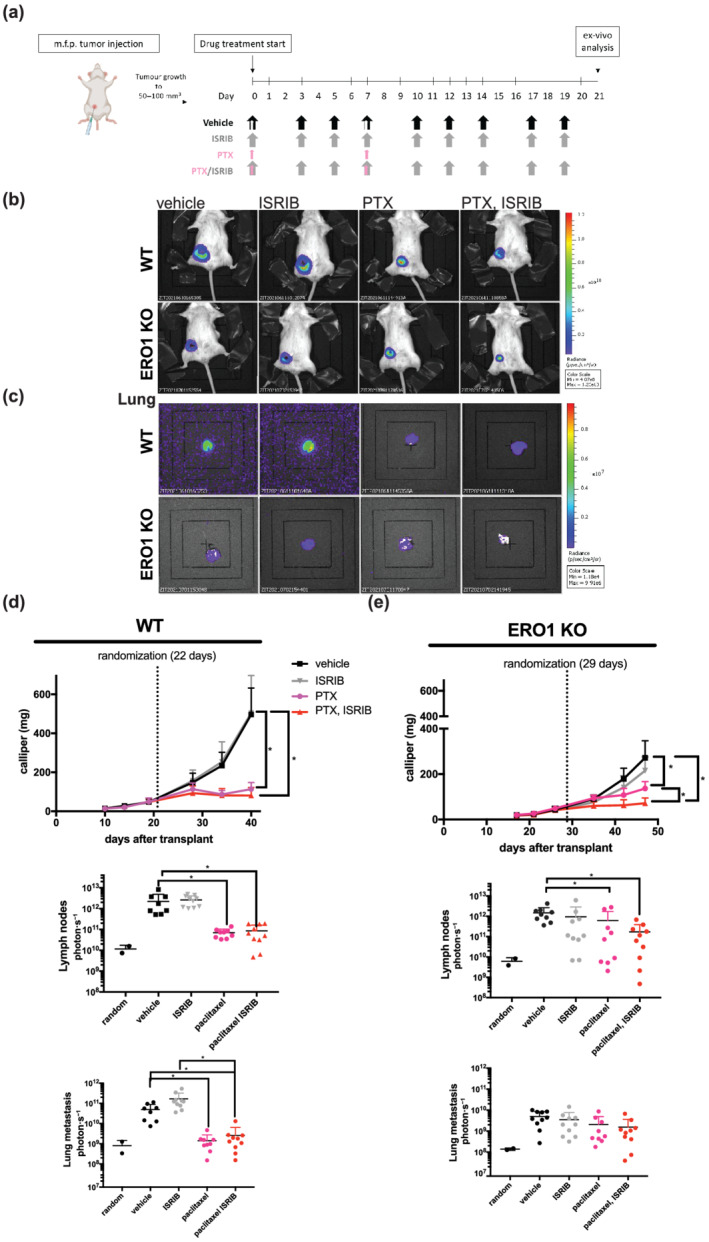
ISRIB together with paclitaxel inhibits tumour growth in ERO1 KO MDAMB231* xenografts. (a) Scheme of the pharmacological treatment of mice injected in the mammary fat pad with WT and ERO1 KO MDA‐MB231*. (b) Bioluminescence signals of primary breast tumours from representative mice (n = 10). (c) Bioluminescence signals of ex vivo lungs. (d) Growth curve of breast tumours measured by the caliper and dot plots on a logarithmic scale of the bioluminescence counts of lymph nodes and lung metastases of WT and (e) of ERO1 KO xenografts.

Mice bearing WT and ERO1 KO MDAMB231* tumours were randomized at 50–100 mm^3^ of breast tumours and treated with vehicle, paclitaxel (15 mg·kg^−1^ IV), ISRIB (2.5 mg·kg^−1^ IP) or paclitaxel plus ISRIB combination therapy (Figure 3a). WT tumours responded well to the cytotoxic effect of paclitaxel, as shown by the arrest of tumour burden and metastases at lymph nodes and lungs, while ISRIB did not improve the efficacy of paclitaxel (Figure 3b–d). ERO1 KO tumours grew more slowly than WT, responded less to paclitaxel, with a tendency to respond to ISRIB, and ISRIB improved the efficacy of paclitaxel (Figure 3b,c,e).

**FIGURE 4:**
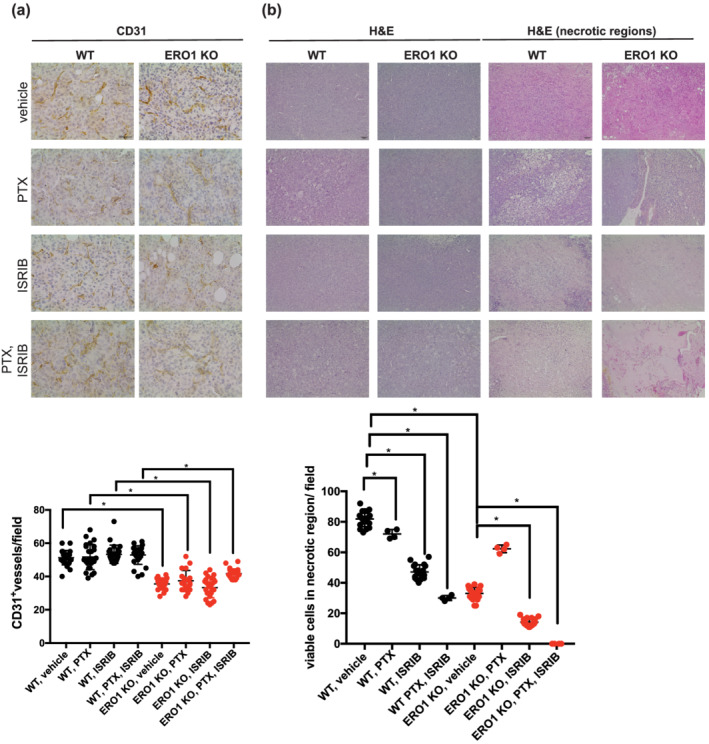
ISRIB does not restrain tumour angiogenesis but reduces cell viability in necrotic areas. (a) Representative micrographs of CD31 IHC staining in primary breast tumours. Below, relative quantification of CD31^+^ blood vessels in random fields (n = 5). (b) Representative H&E (haematoxylin & eosin) staining in primary tumours. On the right, H&E staining of necrotic areas. Below, quantification of the number of viable cells in necrotic areas. Differences were calculated by one‐way ANOVA for multiple comparison tests.

Immunohistological examination of the endothelial markers CD31 and quantification of the related signal confirmed the reduced CD31 staining in ERO1 KO MDAMB‐231* tumours in all treated groups compared with the WT counterparts. These results confirm the impaired angiogenesis in ERO1 KO breast tumours, but also rules out any effect of ISRIB on angiogenesis (Figure 4a). However, H&E staining pointed to reduced necrotic area in ERO1 KO tumours treated with the combination paclitaxel plus ISRIB and suggested that both ISRIB and the combination paclitaxel plus ISRIB reduced cell viability within the necrotic regions of WT and ERO1 KO tumours, with a prominent effect in ERO1 KO tumours (Figure 4b). These findings corroborate our results in ERO1 KO MDA‐MB‐231* cells as well as in breast tumours, pointing to a recovery of ISRIB‐mediated protein synthesis, which impairs a tumour pro‐survival mechanism, but also proving the lack of any effect of ISRIB on tumour angiogenesis.

### 3.4 ERO1 KO breast cancer up‐regulates PERK branch of UPR

To identify pathways that might account for the different responses of WT and ERO1 KO MDAMB231* breast cancer to ISRIB, we used RNA sequencing analysis for the transcriptional profiling of these tumours from mice, which received either ISRIB, or paclitaxel, or the combination of the two drugs.

**FIGURE 5:**
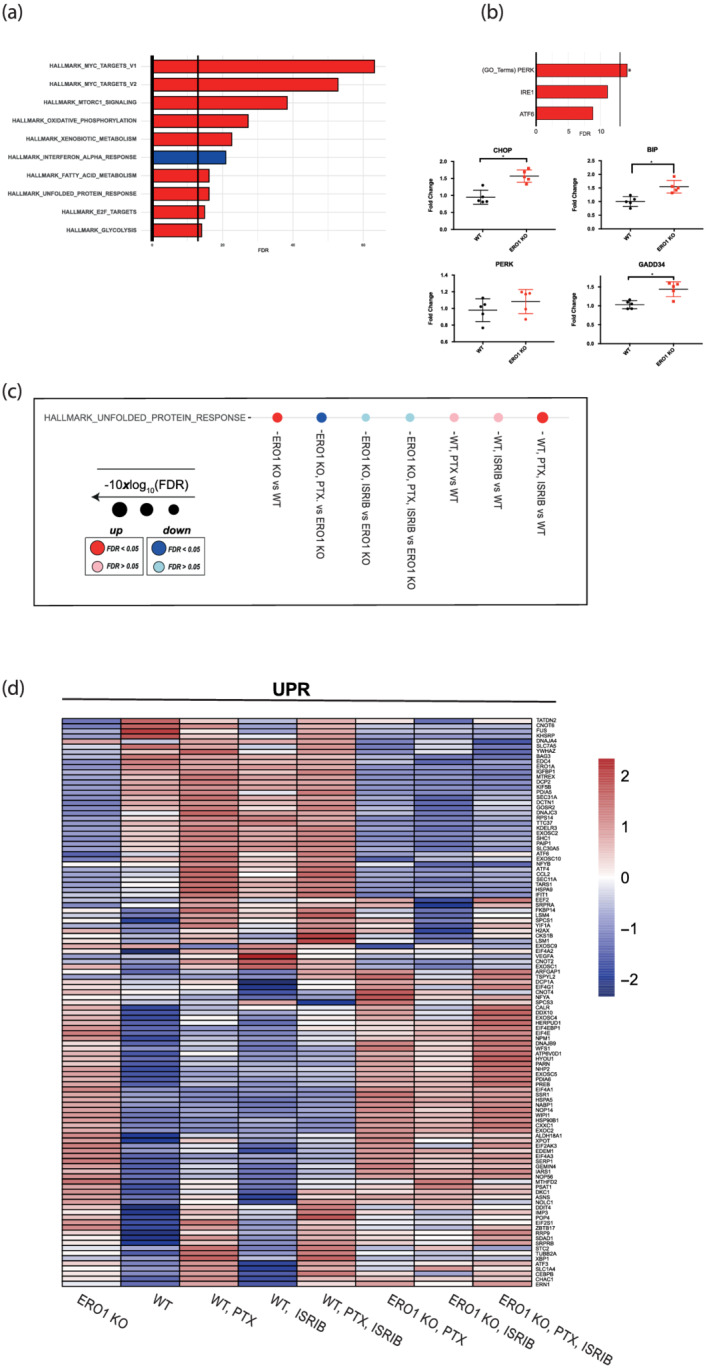
ERO1 KO breast tumours up‐regulate the PERK pathway of the UPR. (a) Bar graphs indicating the top 10 most significantly perturbed gene sets (Hallmark) of ERO1 KO MDAMB231* tumours. Enrichment and their FDR‐adjusted *P*‐values were computed using a camera (pre‐ranked) and were determined on the Hallmark gene sets collection (MSigDB). The X axis reports the logarithmically transformed FDR value in the form of −10xlog10 (FDR), with a bold intercept (X = 13.01) indicating the FDR threshold of 0.05. Red bars: Up‐regulated; blue bars: Down‐regulated. (b) Bar graphs indicating PERK, IRE1, ATF6 gene sets (GO: Gene ontology gene sets) of ERO1 KO MDAMB231* tumours. PERK pathway was up‐regulated in ERO1 KO MDAMB231* tumours. Below, quantitative real‐time PCR on CHOP (*DDIT3*), BIP (*HSPA5*), PERK (*EIF2AK3*), GADD34 (*PPP1R15A*) cDNA from WT and ERO1 KO MDAMB231* tumours (N = 5). (c) Dot plots in Hallmark gene sets indicating the up‐regulation and down‐regulation of UPR in WT and ERO1 KO MDAMB231* tumours from mice given the indicated pharmacological treatments (PTX stands for paclitaxel). (d) Heatmap of UPR genes from the Hallmark gene sets collection in WT and ERO1 KO MDAMB231* tumours.

Data analyses identified genes that were differently regulated in WT and ERO1 KO MDAMB231* breast tumours: 3580 genes decreased, and 3857 genes increased in ERO1 KO tumours. We then ran pathway enrichment analysis (Hallmark gene set) to identify pathways deregulated in ERO1 KO MDAMB231* tumours. Among the top 10 pathways up‐regulated in ERO1 KO tumours compared with their WT counterparts, we found a mammalian/mechanistic target of rapamycin complex 1 (mTORC1) stress signalling, which is part of the integrated response arm of the UPR and involved in activation of protein synthesis (Wouters & Koritzinsky, 2008), and UPR itself (Figure 5a).

Further analysis by GO terms, to distinguish between PERK, IRE1 and ATF6 branches of the UPR, pointed to a selective up‐regulation of PERK pathway in ERO1 KO MDAMB231* tumours. Quantitative real‐time PCR confirmed up‐regulation of *DDIT3* (CHOP), *HSPA5* (BIP), and *PPP1R15A* (GADD34) transcripts belonging to PERK pathway in ERO1 KO MDAMB231* tumours (Figure 5b).

Comparisons of the WT and ERO1 KO tumours and the pharmacological treatments point to different regulation of the UPR pathway in these tumours under ISRIB and paclitaxel (Figure 5c). These treatments induce UPR in WT tumours but down‐regulate UPR in ERO1 KO tumours, suggesting that a different genetic background (i.e. WT vs. ERO1 KO) dictates the regulation on the UPR pathway and paclitaxel and ISRIB act in opposite ways on the UPR regulation of WT and ERO1 KO breast tumours (Figure 5c). The heatmap of Hallmark genes of the UPR pathway confirms the lack of ERO1 in ERO1 KO tumours and points to up‐regulation of different UPR effectors in these tumours. However, ATF4, a pro‐survival effector of the ISR arm of UPR (downstream to the PERK branch), is down‐regulated in the treated ERO1 KO tumours compared with the WT counterparts (Figure 5d). These findings suggest up‐regulation of UPR, and specifically of the PERK arm, in ERO1 KO breast tumours and down‐regulation of UPR after the combination of ISRIB and paclitaxel treatment in the same tumours.

### 3.5 ERO1/PERK in breast cancer patients

Previously we reported that the levels of ERO1 correlate with breast tumour aggressiveness (Varone et al., 2021), whereas others have shown that low PERK levels positively correlate with better overall survival (Jewer et al., 2020).

**FIGURE 6:**
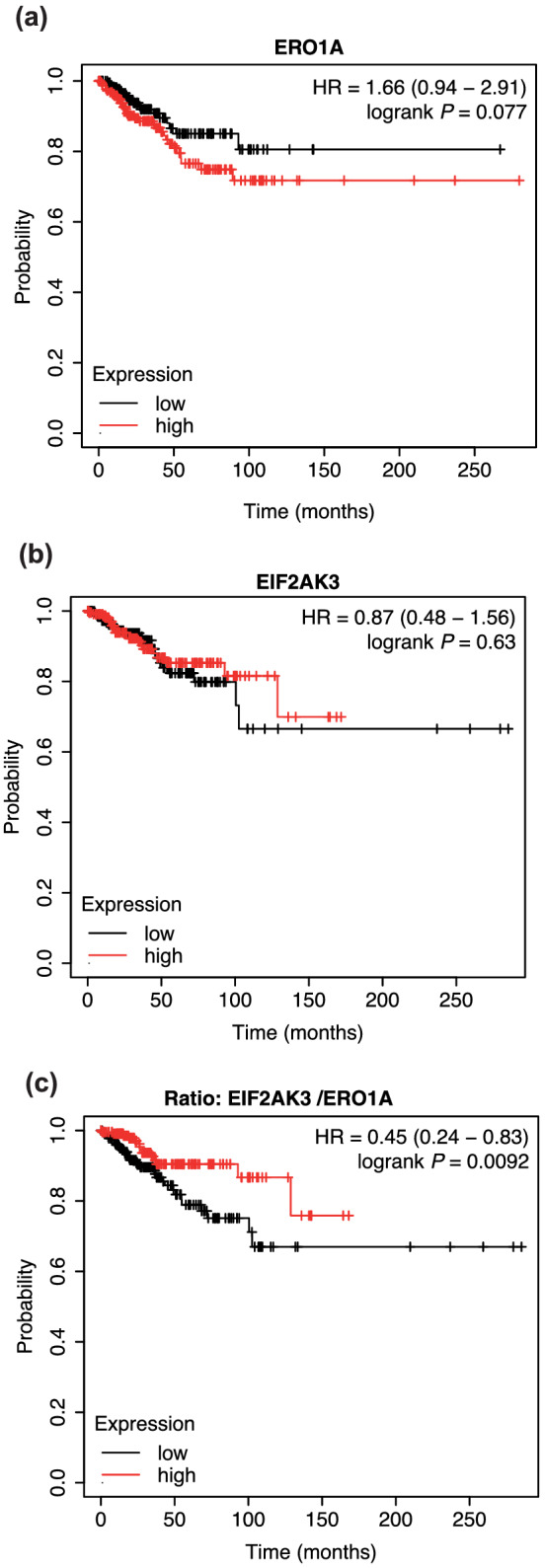
ERO1/PERK cooperation in breast tumours. Kaplan–Meier plotter depicting relapse‐free survival of breast cancer patients (n = 948) stratified for gene expression levels of ERO1 (a), EIF2AK3 (PERK) (b) and the ratio EIF2AK3/ERO1 (c). In panel (c), the upper (n = 237) and lower quartile (n = 237) of the ratio are represented. Statistical significance was assessed using a log‐rank test.

Survival analysis of breast cancer patients, with the KM Plotter tool, indicates no statistically significant difference in terms of overall survival or relapse‐free survival (RFS) when patients were stratified in the upper and lower quartiles for their gene expression levels of ERO1A (Figure 6a) and EIF2AK3 (PERK) (Figure 6b). However, a high EIF2AK3:ERO1A ratio predicts a better outcome, indicating cooperation of PERK with the ERO1 pathway in breast tumours (Figure 6c).

## 4 DISCUSSION

ERO1 is a protein disulphide oxidase that participates in protein oxidative folding of nascent proteins in the endoplasmic reticulum (Zito, 2015). Although its activity in mammals is compensated by other enzymes such as peroxiredoxin (PRDX4), ERO1 deficiency impairs VEGFA folding and secretion in highly aggressive triple‐negative breast tumours (MDAMB231*), curtailing the tumour angiogenesis and metastasis (Varone et al., 2021; Zito, 2013; Zito et al., 2012; Zito, Melo, et al., 2010). ERO1's effect, in its capacity as a protein disulphide oxidase, is not restricted to VEGF but also to other angiogenic factors; thus, the consequence of its deficiency on the restraint of tumour angiogenesis might be highly effective (Manuelli et al., 2021; Varone et al., 2021). Unfortunately, the two ERO1 inhibitors currently available cannot be employed in vivo on account of potential off‐target effects (Blais et al., 2010). Many influential reports have suggested that CHOP regulates ERO1 in disparate ER stress conditions (Li et al., 2009; Marciniak et al., 2004; Pozzer et al., 2018), so we have focused on ISRIB, a small molecule that inhibits the integrated stress response by reactivating protein translation, and also inhibits the downstream CHOP signal (Zyryanova et al., 2021).

In view of the CHOP‐induced ERO1 levels and the beneficial cytotoxic activity of ISRIB in some cancers, we tested whether ISRIB also inhibited ERO1 in preclinical models of breast cancers (Ghaddar et al., 2021; Jewer et al., 2020; Nguyen et al., 2018). We employed triple‐negative breast cancer MDAMB231 cells which, through serial in vivo passages, acquire a more aggressive phenotype in terms of proliferation and metastases, and refer to them as MDAMB231*. After that, we knocked out ERO1 expression (by CRISPR/CAS9 technology), obtaining ERO1 KO MDAMB231*.

WT and ERO1 KO MDAMB231* cells responded differently to hypoxia, which is a common stress condition in solid tumours and their micro‐environment. Indeed, we saw that ERO1 KO cells reduce protein synthesis more effectively by activating the ISR arm of UPR (Leprivier et al., 2015; Wouters & Koritzinsky, 2008).

We therefore tested ISRIB's effects on highly aggressive WT and ERO1 KO MDAMB231* under hypoxic conditions. ISRIB was more effective in reactivating the protein translation in ERO1 KO MDAMB231* under hypoxia, which experience proteotoxicity and therefore, impaired their cell viability. In accordance with a previous report, we confirmed the ability of ISRIB to inhibit CHOP expression (Zyryanova et al., 2021). However, under these conditions, ISRIB does not impair either ERO1 expression or its activity, indicating that ERO1 is not regulated through CHOP in hypoxic breast cancer, and therefore ISRIB only blunts the CHOP signal but not that of ERO1, or the functionally related tumour angiogenesis. This outcome corroborates recent findings, pointing instead to the regulation of ERO1 by the transcription factor nuclear factor IB (NFIB) in breast cancer (Zilli et al., 2021).

Irrespective of this, there was a synergistic effect in restraining tumour growth and metastasis in ERO1 KO MDAMB231*‐bearing mice treated with ISRIB, despite the lack of any significant response in ISRIB‐treated WT‐tumour bearing mice. Importantly, the RNA sequencing data on ERO1 KO MDAMB23* breast cancer indicate an increase in UPR and specifically in ER‐resident kinase PERK (eIF2AK3) branch, suggesting activation of the PERK arm of the UPR.

The PERK pathway connects ER stress to repression of protein translation and, by up‐regulating enzymes and chaperones, fosters protein folding (Walter & Ron, 2011). Our findings highlight an adaptive mechanism whereby the lack of ERO1 in breast tumour cells converges on the PERK pathway of the UPR and, by attenuating protein translation, limits the proteotoxicity. The repression of protein synthesis which, while giving ERO1‐deficient cells a significant pro‐survival benefit, renders them more susceptible to ISRIB.

ISRIB reactivates protein translation by binding eIF2B, a guanine nucleotide exchange factor for eIF2, which becomes resistant to the inhibitory effect of p‐eIF2alpha, and thus weakens the prosurvival effect of PERK‐mediated repression of the protein translation, which results detrimental in the context of the impaired proteostasis imposed by ERO1 deficiency (Han et al., 2013; Zyryanova et al., 2021).

Therefore ERO1 deficiency from one side impairs proteostasis, while from the other side it represses the protein translation through PERK activation, in ER stress conditions relevant for tumours such as hypoxia. Consequently, the low load of protein translation upon ERO1 deficiency predisposes ERO1 KO MDAMB231* breast cancer cells to become susceptible to ISRIB, which is then toxic by increasing protein translation in a context of impaired proteostasis.

The outcome of ISRIB's selective effect on ERO1 KO MDAMB231* breast tumours with up‐regulated PERK pathway is in line with other reports of the effectiveness of ISRIB on mutant KRAS lung cancer with high PERK/p‐eIF2alpha (Ghaddar et al., 2021; Jewer et al., 2020). These findings suggest that activation of the PERK arm of the UPR, imposing low levels of protein translation together with impaired proteostasis, is a prerequisite for the cytotoxic effect of ISRIB on cancer cells.

Resistance to paclitaxel, one of the first‐line drugs for breast cancer, was suggested to be due to UPR (Lee et al., 2011). This proposal might suggest that a resistance to paclitaxel may be counteracted by drugs that weaken UPR. Our RNA sequencing data suggest an opposite response of WT and ERO1 KO breast cancer to the combination of ISRIB and paclitaxel on the UPR pathway: UPR is up‐regulated in WT tumours treated with the combination, but is down‐regulated in ERO1 KO tumours treated with the same pharmacological combination. These different UPR responses of WT and ERO1 KO tumours to the combination paclitaxel and ISRIB, together with the impairment of ERO1 KO tumour burden, indicate that UPR weakening correlates with a cytotoxic response of cancer to these two drugs.

**FIGURE 7:**
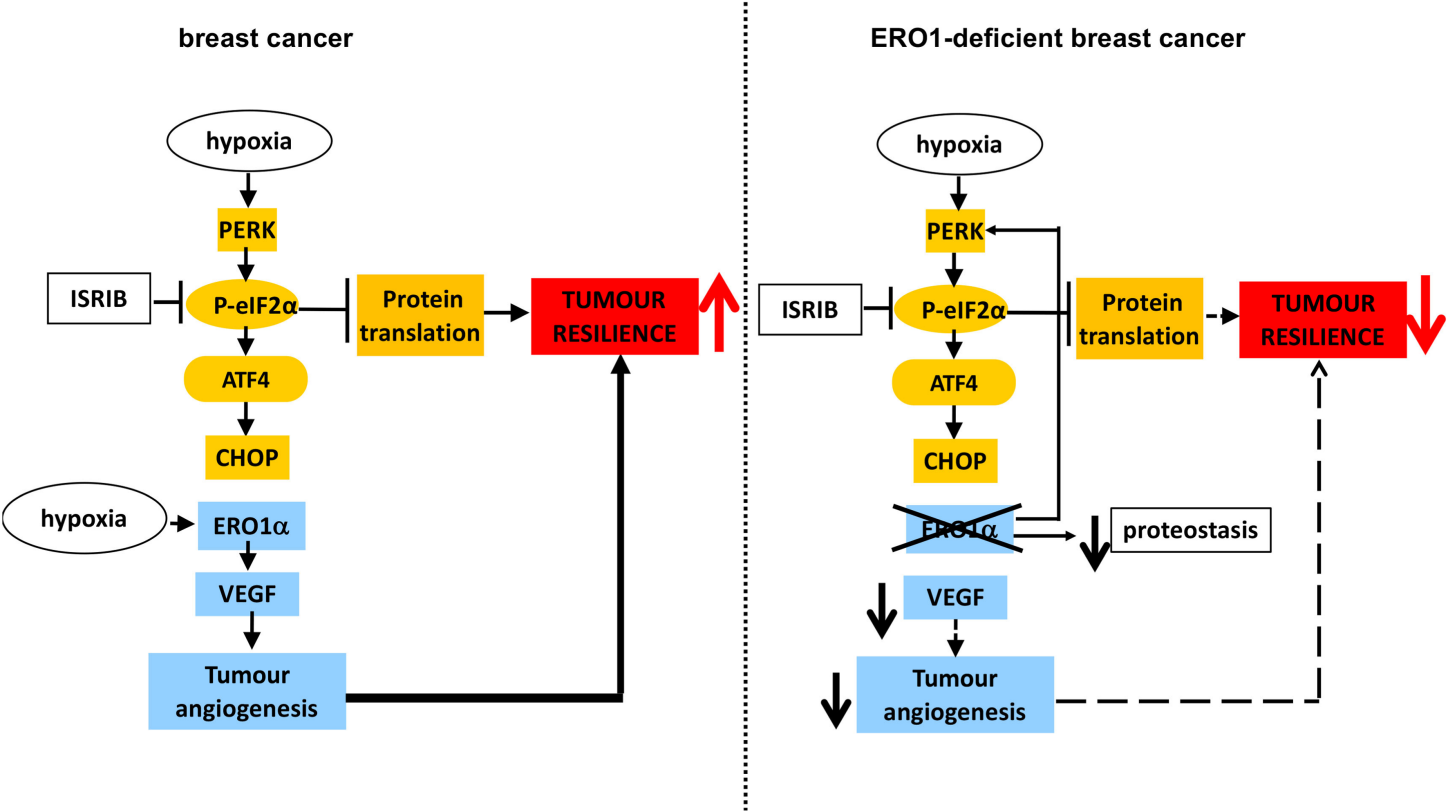
ERO1 deficiency in breast tumours up‐regulates PERK and dictates the ISRIB‐mediated cytotoxic effect. ERO1 is a protein disulphide oxidase in the endoplasmic reticulum, whose expression is regulated by CHOP in a variety of ER stress conditions. Previously, we reported that the lack of ERO1 in highly metastatic breast tumours impairs secretion of angiogenic factors, among which VEGFA, and angiogenesis, hence acting on the tumour resilience. In this study, we employed ISRIB, a small molecule which, by reactivating protein translation, enfeebles the adaptive PERK‐mediated mechanism of protein repression. In breast tumour (MDAMB231*) cells under hypoxia, ISRIB inhibits CHOP but has no effect on ERO1 activity, suggesting that under hypoxia CHOP does not regulate ERO1. However, ISRIB is synergistic with ERO1 deficiency in terms of impairment of the tumour burden. Mechanistically, ERO1 deficiency up‐regulates the PERK branch of UPR, repressing protein translation, which renders ISRIB more effective to restrain tumour growth in a context of impaired proteostasis. In ERO1 KO tumours, ISRIB‐dependent reactivation of protein translation together with the impairment of angiogenesis constitutes a double‐hit which weakens tumour resilience to stress.

In conclusion, our study supports the notion that the PERK arm of UPR with the downstream attenuation of protein translation is an important adaptive mechanism of tumourigenesis and that ISRIB impairs this mechanism of cancer adaptation by reactivating protein translation. Furthermore, our findings on ERO1‐deficient breast tumours suggest that ISRIB restrains the growth of tumours with high PERK and a low load of protein translation/low ERO1, possibly because the rapid ISRIB‐dependent increase in protein translation results in a toxic action in cells deficient in an enzyme with protein folding activity, hence, with impaired proteostasis (Figure 7). Our analysis of breast tumour patients indicated better relapse‐free survival of those with a high PERK/ERO1 ratio, through a cooperation between ERO1 and the PERK pathway in these tumours. To conclude, ISRIB may be a valid drug in the pharmacological armamentarium for breast tumours with high PERK and low ERO1 levels and, thus impaired proteostasis.

## CONFLICT OF INTERESTS

The authors declare no competing interests.

## AUTHOR CONTRIBUTIONS

EV and AD conducted the experiments. MCB, MB, and LD performed library preparation and next generation sequencing. FP conducted histochemical staining. RG designed in vivo experiments. EZ acquired funding, designed and oversaw the experiments, and wrote the manuscript.

## DECLARATION OF TRANSPARENCY AND SCIENTIFIC RIGOUR

This Declaration acknowledges that this paper adheres to the principles for transparent reporting and scientific rigour of preclinical research as stated in the *BJP* guidelines for Research, Design and Analysis, Immunoblotting and Immunochemistry, and Animal Experimentation, and as recommended by funding agencies, publishers and other organizations engaged with supporting research.

## Supporting information


**Figure S1** Representative immunoblot of p‐eIF2alpha and the total eIF2alpha in protein lysates from WT and ERO1 KO MDAMB231* under normoxic and hypoxic conditions and treatment with the ER stress inducer thapsigargin. Actin was used as a loading control. Tg stands for thapsigargin that was used to treat cells at a concentration of 0.5 micromolar for 4 and 6 h to induce phosphorylation of eIF2alpha. On the right, dot plots indicating phosphorylation of eIF2‐alpha on the total eIF2‐alpha in hypoxic conditions and after Tg treatment of WT and ERO1 KO cells. The ratio eIF2‐alpha on the total eIF2‐ alpha was set at 1 for WT and ERO1 KO cells in normoxic conditions (n = 6).
**Figure S2** A) Quantitative real‐time PCR on cDNA from WT and ERO1 KO MDAMB231* cells (n = 6). B) Quantitative real‐time PCR on cDNA from parental WT MDAMB231 (a less aggressive cell line than the in vivo transformed MDAMB231*) and MCF7 (a luminal cell line) (n = 6).

## Data Availability

The datasets used and/or analysed during the current study are available from the corresponding author on reasonable request. Raw RNA‐sequencing data have been deposited in European Nucleotide Archive under the accession E‐MTAB‐11313.
