# Supplementary material for: Endoplasmic reticulum oxidoreductin 1‐alpha deficiency and activation of protein translation synergistically impair breast tumour resilience
Source: Br J Pharmacol. 2022 Aug 11;179(23):5180–95. doi: 10.1111/bph.15927 (PMC9804893; doi:10.1111/bph.15927)

Figure Supplementary 1

Representative immunoblot of p-eIF2alpha and the total eIF2alpha on protein lysates from WT and ERO1 KO MDAMB231* under normoxic and hypoxic conditions and treatment with the ER stress inducer thapsigargin. Actin was used as a loading control. Tg stands for thapsigargin that was used to treat cells at a concentration of 0.5 micromolar for 4 and 6h to induce phosphorylation of eIF2alpha. On the right, dot plots indicating phosphorylation of eIF2-alpha on the total eIF2-alpha in hypoxic conditions and after Tg treatment of WT and ERO1 KO cells. The ratio eIF2-alpha on the total eIF2-alpha was set at 1 for WT and ERO1 KO cells in normoxic conditions (N=6).

Figure Supplementary 2

A) Quantitative real-time PCR on cDNA from WT and ERO1 KO MDAMB231* cells (N=6). B) Quantitative real-time PCR on cDNA from parental WT MDAMB231 (a less aggressive cell line than the in vivo transformed MDAMB231*) and MCF7 (a luminal cell line) (N=6).

Figure Supplementary 1

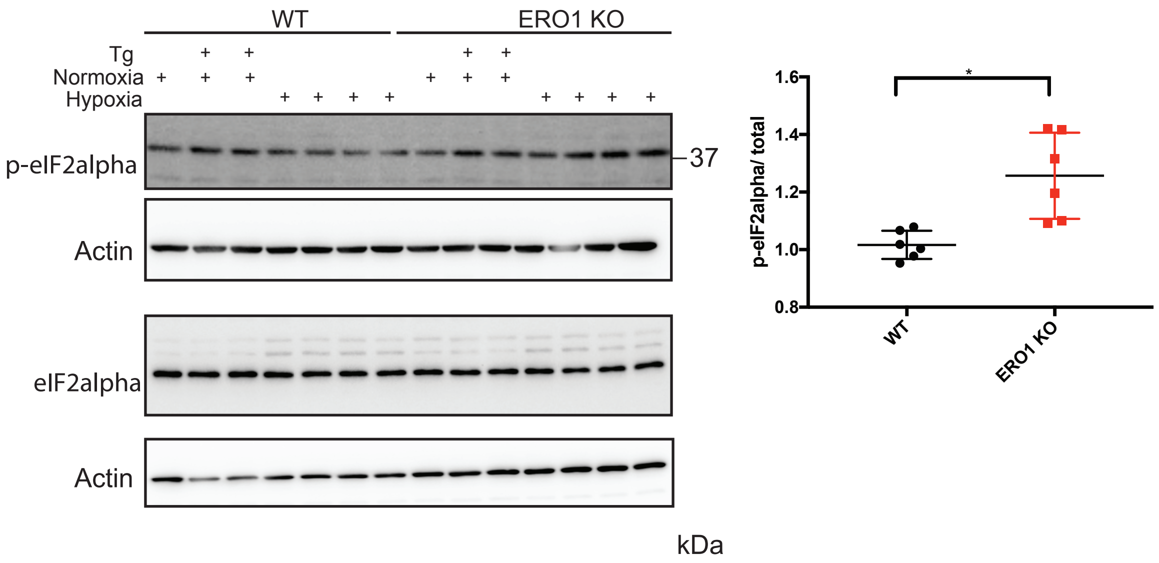

Figure Supplementary 2

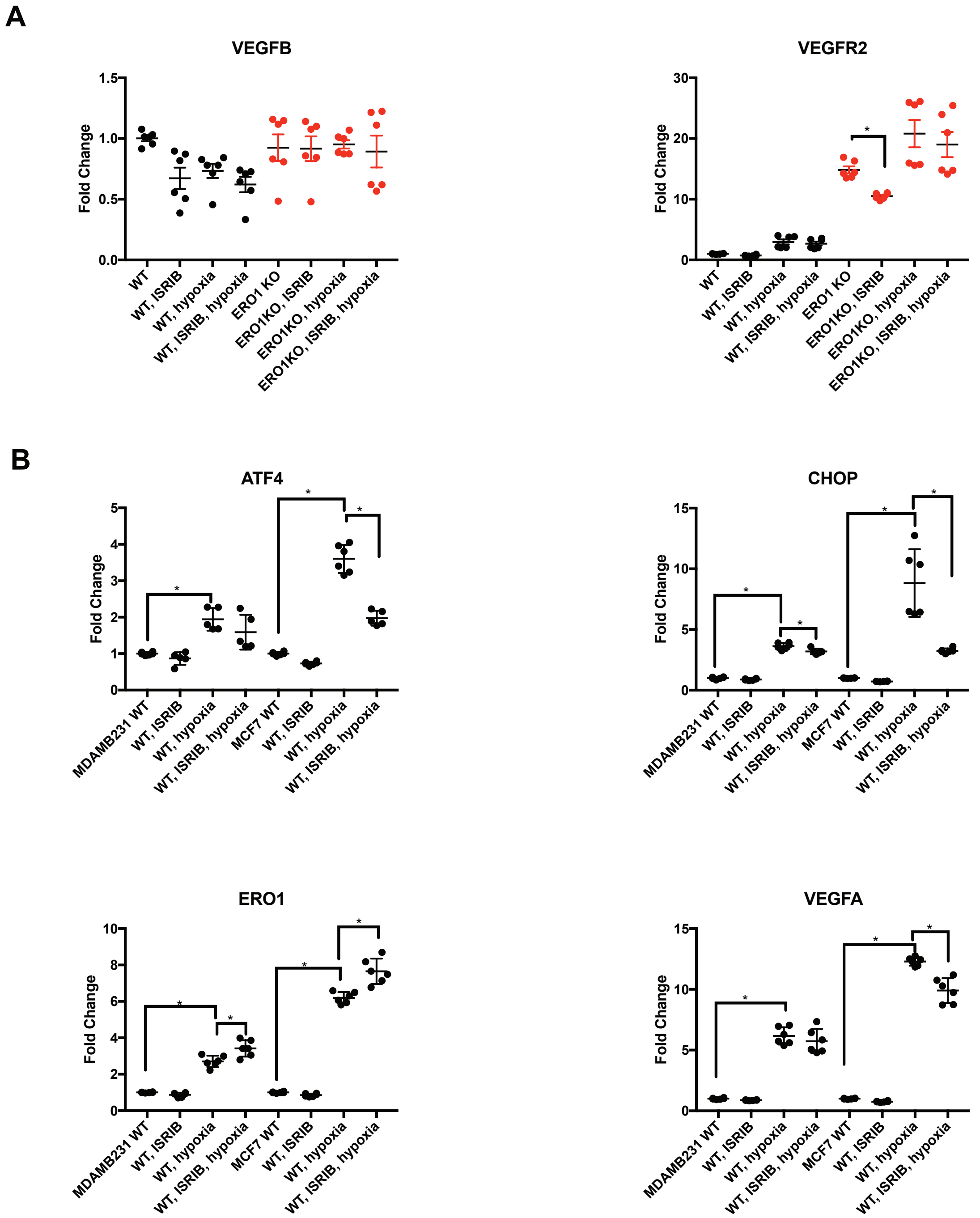

Supplement: Supplementary file 1 — Figure S1 Representative immunoblot of p‐eIF2alpha and the total eIF2alpha in protein lysates from WT and ERO1 KO MDAMB231* under normoxic and hypoxic conditions and treatment with the ER stress inducer thapsigargin. Actin was used as a loading control. Tg stands for thapsigargin that was used to treat cells at a concentration of 0.5 micromolar for 4 and 6 h to induce phosphorylation of eIF2alpha. On the right, dot plots indicating phosphorylation of eIF2‐alpha on the total eIF2‐alpha in hypoxic conditions and after Tg treatment of WT and ERO1 KO cells. The ratio eIF2‐alpha on the total eIF2‐ alpha was set at 1 for WT and ERO1 KO cells in normoxic conditions (n = 6). Figure S2 A) Quantitative real‐time PCR on cDNA from WT and ERO1 KO MDAMB231* cells (n = 6). B) Quantitative real‐time PCR on cDNA from parental WT MDAMB231 (a less aggressive cell line than the in vivo transformed MDAMB231*) and MCF7 (a luminal cell line) (n = 6). [file BPH-179-5180-s001.pdf]
